# Fragmentation of Human Neutrophil α-Defensin 4 to Combat Multidrug Resistant Bacteria

**DOI:** 10.3389/fmicb.2020.01147

**Published:** 2020-06-03

**Authors:** Dirk Ehmann, Louis Koeninger, Judith Wendler, Nisar P. Malek, Eduard F. Stange, Jan Wehkamp, Benjamin A. H. Jensen

**Affiliations:** ^1^Department of Internal Medicine I, University Hospital Tübingen, Tübingen, Germany; ^2^Faculty of Health and Medical Sciences, Novo Nordisk Foundation Center for Basic Metabolic Research, Human Genomics and Metagenomics in Metabolism, University of Copenhagen, Copenhagen, Denmark

**Keywords:** host defense peptides, α-defensins, proteolytic digestion, multidrug resistance, HNP-4

## Abstract

The occurrence and spread of multidrug-resistant bacteria is a prominent health concern. To curb this urgent threat, new innovative strategies pursuing novel antimicrobial agents are of the utmost importance. Here, we unleashed the antimicrobial activity of human neutrophil peptide-4 (HNP-4) by tryptic digestion. We identified a single 11 amino acid long fragment (HNP-4_1__–__11_) with remarkable antimicrobial potential, exceeding that of the full length peptide on both mass and molar levels. Importantly, HNP-4_1__–__11_ was equally bactericidal against multidrug-resistant and non-resistant strains; a potency that was further enhanced by N- and C-terminus modifications (acetylation and amidation, respectively). These observations, combined with negligible cytotoxicity not exceeding that of the full length peptide, presents proteolytic digestion of innate host-defense-peptides as a novel strategy to overcome the current health crisis related to antibiotic-resistant bacteria.

## Introduction

The spread and occurrence of new multidrug-resistant bacteria represents a prominent and emerging health care threat on a global scale. At large, the pharmaceutical industry and governments alike have failed to develop new antibiotics which has urged World Health Organization (WHO) to call out for new cost-effective strategies to fight these devastating pathogens (Tacconelli et al., 2018). A major challenge is the divergent motivation from society vs. companies, where novel strategies are welcomed yet shelved by regulatory authorities. The rationale behind such decisions, mitigating the risk of multidrug resistance while ensuring these novel therapies in case of an outbreak, is justified, yet jeopardizes the costly development of novel antibiotics. Thus, new cost-effective strategies more resilient to multidrug resistance are urgently needed (Sukkar, 2013; Falagas et al., 2016). Host-defense-peptides (HDP) – previously known as antimicrobial peptides (AMPs) – possess a broad range of antimicrobial properties, which could be useful to develop new antimicrobials in the fight against resistant pathogens (Zasloff, 2002). Defensins are the most prominent class of HDPs in humans. These small cationic molecules share as a common motive six conserved cysteines, which from three disulfide bonds classifies them into α- and β-defensins (White et al., 1995; Ganz, 2003; Selsted and Ouellette, 2005). Four of the six human α-defensins are expressed by immune cells, namely human neutrophil peptides 1–4 (HNPs), whereas the remaining two, human α-defensin 5 and 6 (HD-5 and HD-6) are expressed by Paneth cells in the small intestine (Lehrer and Lu, 2012). All HNPs are processed from propeptide to mature form during their trafficking activated by proteolytic digestion in polymorphonuclear neutrophils azurophilic granules (Valore and Ganz, 1992). These granules fuse with the lysosome after phagocytosis of pathogens allowing for context specific bactericidal activity (Ganz et al., 1985; Selsted et al., 1985). Based on the biological control of these processes it is hypothesized that synthetic production of said peptides could be used as an antibiotic tool against extracellular pathogens. Yet, large-scale expression of accurately folded defensins is a major cost-challenge. Inspired by our recent observation that duodenal fluid degrades full length HD-5 to multiple biological active fragments with different antimicrobial properties including potency, efficacy and bacterial spectrum (Ehmann et al., 2019), we hypothesized that enzymatic digestion of mature HDPs could unleash their antimicrobial capacity and concomitantly solve the production-cost challenge of full length peptides. To this end, the least expressed HNP, HNP-4 (Harwig et al., 1992; Hu et al., 2019), is more bactericidal against Gram-negative bacteria than any of HNP-1-3 (Ericksen et al., 2005). While HNP-1-3 only differs internally in the first amino acid sequence, HNP-4 is more divergent combined with an increased negative charge ultimately enhancing antimicrobial activity (Lehrer and Lu, 2012). We used HNP-4 as precursor to identify new therapeutic agents. To this end, tryptic digestion of the linearized full length peptide liberated its antimicrobial potential. We identified a single fragment with a remarkable bactericidal potency, exceeding the MIC of the full length peptide on molar level. Surprisingly, we observed the antimicrobial efficacy of said peptide to be equally efficient against multidrug-resistant and non-resistant strains, hence presenting HDP fragmentation (Latendorf et al., 2019) as an innovative and cost-effective strategy to aid curbing the emerging threat of antibiotic resistance.

## Materials and Methods

### Bacterial Strains

*B. adolescentis* Ni3,29c and *B. breve* were provided by Ardeypharm GmbH (Herdecke, Germany). *L. rhamnosus* GG was obtained from InfectoPharm Arzneimittel and Consilium GmbH (Heppenheim, Germany). *A. baumannii* DSM30007, *B. vulgatus* DSM1447, *E. coli* MC1000 DSM6214, *E. coli* DSM8695 (EPEC), *E. coli* DSM10729 (UPEC), *E. faecalis* DSM20478, *E. faecium* DSM20477, *K. pneumoniae* DSM30104, and *S. epidermidis* DSM20044 were obtained from Deutsche Sammlung von Mikroorganismen und Zellkultur GmbH (Braunschweig, Germany). *A. baumannii* 4-MRGN, *B. longum*, *E. coli* ATCC25922, *E. faecium*, *E. faecalis ATCC29212*, *K. pneumoniae* 3-MRGN, *L. fermentum*, *L. salivarius*, *P. aeruginosa* ATCC27853, *P. aeruginosa* 4-MRGN, *S. enterica* serovar Enteritidis, *S. aureus* ATCC25923 *and S. salivarius* were obtained as clinical isolates from the Robert-Bosch-Hospital Stuttgart, Germany. *B. subtilis* (trpC2), *E. coli* JM83, *P. aeruginosa* PAO1, *P. aeruginosa* XPAT1, *P. aeruginosa* XPAT2, *S. aureus* USA300 and *Y. enterocolitica* were provided by the Interfaculty Institute for Microbiology and Infection Medicine, Tübingen, Germany.

### Peptides

HNP-4 (Purity ≥ 99%) was obtained from PeptaNova GmbH (Sandhausen, Germany). All peptide fragments, HNP-4_1__–__11_ and HNP-4_1__–__11__mod_ were chemically synthesized by EMC Microcollections GmbH (Tübingen, Germany) and purified by precipitation. EMC Microcollections guarantees a purity >> 90% by HPLC analysis (Supplementary Figure S3). All peptides were dissolved in 0.01% acetic acid.

### Screening for Fragments of HNP-4 Using LC/MS

As previously described (Ehmann et al., 2019), 2.5 μg of HNP-4 were incubated in 50 mM NH_4_HCO_3_ buffer (pH 8.0; Fluka) with 2 mM *tris* (2-carboxyethyl) phosphine for 15 min at 37°C. Afterward, 0.05 μg trypsin [1:50 (w/w)] was added and incubated for additional 30 min at 37°C. Lastly, formic acid and acetonitrile in a final concentration of 0.5 and 10% were added, respectively, and the samples analyzed by mass spectrometry. Mass spectrometry was performed as a LC/MS system using an Agilent 1200 series HPLC with an Agilent Advanced Bio Peptide Map (2.1 × 150 mm, 2.7 μm) column with a flow of 0.4 ml/min at 55°C column temperature and a 6540 UHD Q-TOF LC/MS system (Agilent) for mass analysis. The samples were separated by a gradient of acetonitrile in 0.1% formic acid. The gradient started at 2% acetonitrile for 4 min and then increases during 35 min to 45%. Mass spectrometric analyses were performed in single MS mode from 100 to 3400 m/z with positive ion polarity and were analyzed by Agilent MassHunter Quantitative Analysis B 06.00 software.

### Screening for Potential Dimers of HNP-4_1__–__11_ and HNP-4_1__–__11__mod_ Using HPLC-MS

To analyze possible inter-/intramolecular dimer formation HPLC-MS were performed by EMC Microcollections GmbH Tübingen. HPLC-MS was performed using a Chromolith Fast Gradient RP18e, 50 × 2 mm column (Merck) with detection at a wavelength of 214 nm, followed by an ESI-MS analysis. The samples were separated by a gradient of MeCN (acetonitrile) containing 0.1% FA (monofluoroacetic acid) from 0 to 100% in 30 min.

### Radial Diffusion Assay

Antimicrobial activity of all peptides was assessed with a modified version of the radial diffusion assay as described earlier (Schroeder et al., 2011b). Briefly, bacteria were cultivated (anaerobic bacteria in anaerobic jars with AnaeroGen, Oxoid, United Kingdom) for up to 18 h in liquid TSB medium. Log-phase bacteria were washed with 10 mM sodium phosphate buffer; pH 7.4 and diluted to 4 × 10^6^ CFU/ml in 10 ml agar (10 mM sodium phosphate buffer, pH 7.4 with 0.3 mg/ml TSB powder and 1% (w/v) low EEO-agarose (AppliChem). Bacteria were incubated under aerobic or anaerobic conditions, respectively, with 2 μg HNP-4 or 4 μg of each fragment for 3 h at 37°C. Afterward, plates were covered with 10 ml of an overlay-gel containing 6% (w/v) TSB powder, 1% (w/v) agar and 10 mM sodium phosphate buffer and incubated for 24 h. The diameter of the inhibition zones corresponds to the antimicrobial activity, when subtracting the diameter of 2.5 mm corresponding to the diameter of the punched well. Experiments were repeated at least three times.

### Turbidity Broth Assay

Log-phase bacteria were washed twice with 10 mM sodium phosphate buffer containing 1% (w/v) TSB. Approximately 4 × 10^5^ CFU/ml bacteria were incubated with serial peptide concentrations (1.56–100 μM) in a final volume of 100 μl in 10 mM sodium phosphate buffer containing 1% (w/v) TSB for 2 h at 37°C. Afterward, 100 μl of 6% TSB (w/v) were added and absorbance was measured at 600 nm (Tecan, Switzerland) and monitored for 12 h. Experiments were carried out at least three independent times.

### Time-Kill Assay

Log-phase bacteria (5 × 10^5^ CFU/ml) were incubated with 6.25 μM of HPN-4_fl_, HNP-4_1__–__11_, HNP-4_1__–__11mod_ or 0.01% acetic acid as a control in 10 mM sodium phosphate buffer containing 1% (w/v) TSB. After incubation at 37°C and 150 rpm for 0 to 120 min, a sample was taken from the suspension and added to a 0.05% (v/v) sodium polyanethole sulfonate (Sigma-Aldrich) solution, which neutralizes remaining peptide activity, and plated on LB agar to determine the number of viable bacteria. Experiments were carried out at least three independent times.

### Reduction Assay

The amino acid sequences of HNP-4_1__–__11_ and HNP-4_1__–__11__mod_ contain cysteines which might form disulfide bonds with another fragment. As reducing agent Dithiothreitol (DTT) was used. Both peptides, HNP-4_1__–__11_ and HNP-4_1__–__11__mod_ were pre-incubated with either 0.1 mM or 1 mM DTT for 1 h at room temperature followed by a turbidity broth assay with ~4 × 10^5^ CFU/ml bacteria as described above. The MIC of HNP-4_1__–__11_ and HNP-4_1__–__11__mod_ was determined against different bacteria strains. Experiments were carried out at least three independent times.

### Protease Inhibitor Assay

Log-phase bacteria were cultivated for up to 18 h in TSB containing different concentrations (0.01 or 0.1) of Bacterial ProteaseArrest^TM^ (G-Biosciences) and 0.5 M EDTA. Bacteria were washed with twice with 10 mM sodium phosphate buffer containing 1% (w/v) TSB and the optical density at 600 nm was adjusted to 0.1. Approximately 5 × 10^5^ CFU/ml bacteria were incubated with serial peptide concentrations (1.56–12.5 μM) in a final volume of 100 μl in 10 mM sodium phosphate buffer containing 1% (w/v) TSB and (0.01 or 0.1) of Bacterial ProteaseArrest^TM^ and 0.5 M EDTA for 2 h at 37°C. After incubation, 100 μl of 6% TSB (w/v) were added and absorbance was measured at 600 nm (Tecan, Switzerland) and monitored for 12 h. Experiments were carried out at least three independent times.

### Cell Toxicity Assay

Experiments were conducted with the human colonic epithelial adenocarcinoma cell line CaCo2 subclone TC7 which was obtained from the Robert-Bosch-Hospital Stuttgart, Germany. HT29 MTX cells subclone E12 (Merck, Germany) were used as an additional colorectal carcinoma cell line. Cells were used at an internal early passage of about 25–40. For experiments, 1500 cells/well were seeded in a 96-well plate in 90 μl media.

Cells were treated with serial peptide concentrations (1.56–100 μM) in a final volume of 100 μl and incubated for 96 h. Afterward, the CellTiter-Glo^®^ 2.0 Cell Viability Assay (Promega, United States) was performed based on the company’s protocol. Experiments were carried out at least three independent times.

### Hemolytic Activity of HNP4 Fragments

Hemolytic activity assay was performed as described earlier (Oddo and Hansen, 2017). Briefly, 1 ml O neg whole blood was washed twice with PBS, centrifuged and 1% (v/v) erythrocytes suspension prepared. Erythrocytes were incubated with serial peptide concentrations (1.56–100 μM) for 1 h at 37°C. Then, samples were centrifuged, supernatant collected and optical density measured at 414 nm. Toxicity against erythrocytes was relative determined to the hemolytic activity of 0.1% Triton X-100. Experiments were carried out in duplicates and performed twice.

### Ethics Statement

The study protocol was previously approved by the Ethical Committee of the University Hospital Tübingen, Germany. Patients and controls who were included in this study all gave their written and informed consent after the study purpose, samples procedure, and potential adjunctive risks were explained. All experiments were conducted in accordance with the relevant guidelines and regulations.

## Results

### Identification of a Novel HNP-4 Fragment After Tryptic Digestions

**FIGURE 1:**
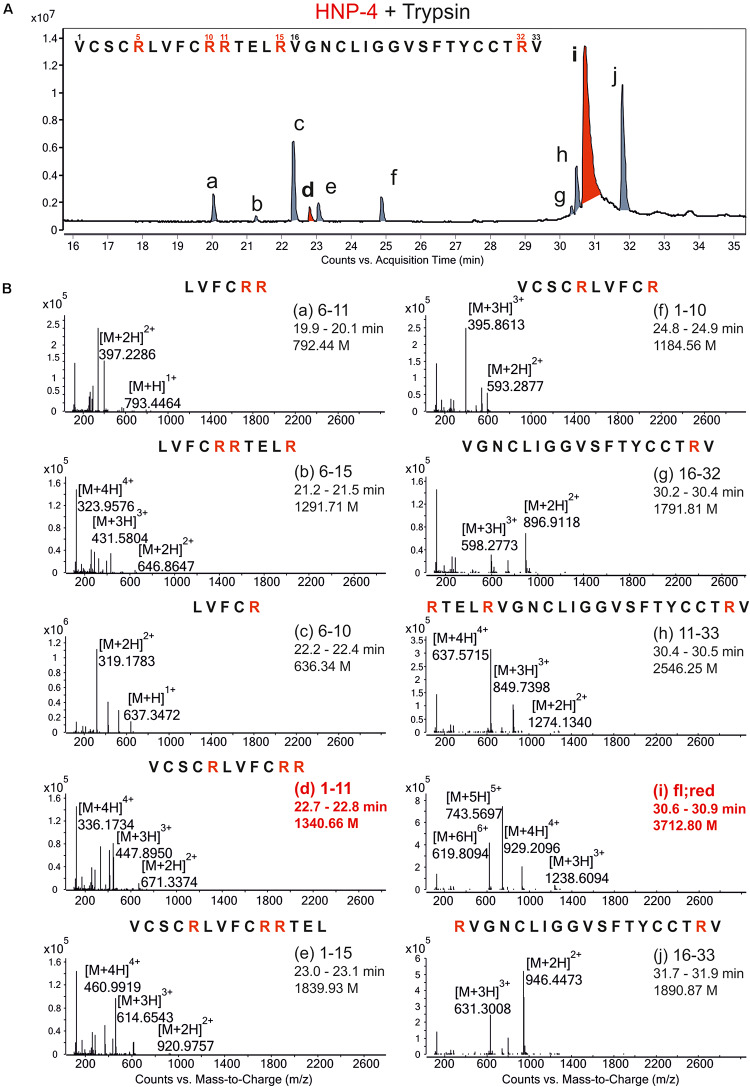
Proteolytic digestion of reduced HNP-4 by trypsin produced different fragments. **(A)** Displays an overview of the chromatogram from an incubation of reduced HNP-4 with trypsin after reduction with 2 mM TCEP. All detectable fragments were marked in red or gray (a–j) and listed due to their retention time. Panel **(B)** show the mass-to-charge (m/z) graphs of all detected fragments. In all mass-to-charge graphs we pointed out the neutral mass based on the detected ions. All peptides marked in red were chose for synthesis and further investigations.

To generate possible fragments out of HNP-4 we used trypsin as a serine protease. It is known from previous work that folded defensins seemed to be stable against proteolytic digestion (Schroeder et al., 2011a). We incubated HNP-4 with 2 mM TCEP (tris(2-carboxyethyl)phosphine; Sigma-Aldrich) to open the disulfide bonds leading to a more linear structure susceptible to proteolytic digest. We analyzed the trypsin-incubated reduced HNP-4 via LC/MS methods and were able to detect several fragments according to the observed ions and their mass to charge ratio (Figure 1A). Identified fragments were mostly located in the N-terminal region based on the cleaving sites of trypsin (Figure 1B). As it is commonly accepted that the net charge of AMPs could play an important role to their antimicrobial activity, we focused on HNP-4_1__–__11_ with a positive net charge of +3 Figure 1B, marked in red.

### Antimicrobial Efficacy of HNP-4_1__–__11_ and HNP-4_1__–__11__mod_

**FIGURE 2:**
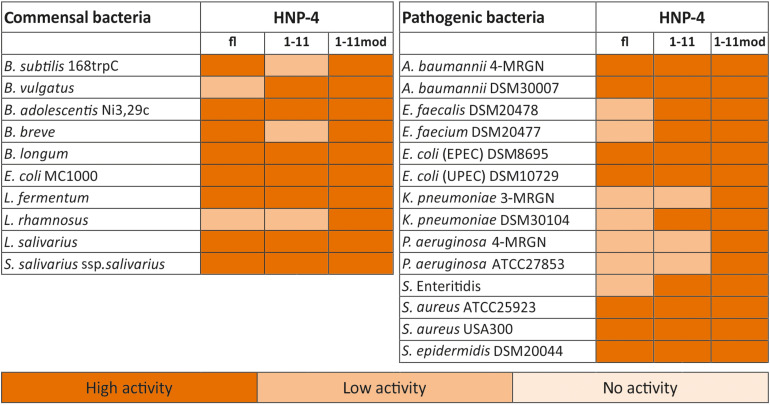
HNP4-derivates display a high antimicrobial activity against commensal and pathogenic bacteria. We analyzed the antimicrobial potential of the identified fragment and its modified version against commensal and pathogenic bacteria. In this heat map, we listed all bacteria and the activity of the fragments in RDA against them. We used 2 μg of the full-length peptide and 4 μg of each fragment. An inhibition zone greater than 8 mm was determined as highly active, between 2.5 and 8 mm as low active, while a diameter of 2.5 mm (diameter of the punched well) was marked as no activity. The heat map is based on three independent experiments.

**FIGURE 3:**
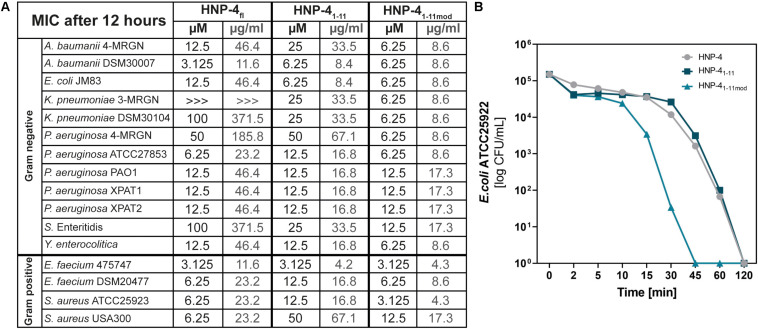
Comparison of the potency (MIC) and efficacy (killing rate) of HNP-4_fl_, HNP-4_1__–__11_ and HNP-4_1__–__11__mod_. **(A)** The minimal inhibitory concentration (MIC) in μM and μg/ml as a concentration without any bacterial growth. Peptides were incubated with tested bacteria and changes in optical density (OD_600_) were measured after 12 h at 37°C. If we were able to observe an antimicrobial effect but did not detect a total inhibition of bacterial growth we marked it with “>>>.”Each experiment was carried out three independent times. **(B)** Killing of *E. coli* ATCC25922 after 0–120 min exposure to 6.25 μM (1× MIC) HNP-4_fl_, HNP-4_1__–__11_ and HNP-4_1__–__11__mod_. Results are expressed as the number of viable bacteria (in log10 CFU) per milliliter. Values are means of three independent experiments.

The natural *in vivo* stability of short linear peptides is generally weak; we therefore used an additional modified form of HNP-4_1__–__11_ (HNP-4_1__–__11__mod_). Here we exchanged the L-amino acids with D-amino acids and modified the N-terminus (acetylation) and C-terminus (amidation). Both modifications should result in a gain of stability (Brinckerhoff et al., 1999; Hong et al., 1999), hence potentially leading to a stronger antimicrobial activity. To analyze the antimicrobial activity of HNP-4_fl_, HNP-4_1__–__11_, and HNP-4_1__–__11__mod_ we used RDAs against a subset of different commensal and pathogenic bacteria (Supplementary Figures S1, S2). All of our tested peptides showed an antimicrobial activity against tested bacteria (Figure 2). While the RDA is the suitable assay to determine a general antimicrobial activity of different peptides, a comparison between different peptides is not possible according to their different abilities (like diffusion) in an agarose gel. We therefore next used a turbidity broth assay to determine the minimal inhibitory concentration (MIC) of HNP-4_fl_, HNP-4_1__–__11_, and HNP-4_1__–__11__mod_ against pathogenic (some multidrug-resistant) Gram negative and positive bacteria (Figure 3A). While all peptides displayed antimicrobial activity against tested bacteria (sole exception: HNP-4_fl_ against *K. pneumoniae* DSM30104), HNP-4_1__–__11_ was surprisingly equimolar to HNP-4_fl_, indicating that the antimicrobial potency of the natural complex-to-produce HNP-4_fl_ is chiefly driven by the first 11 amino acids (HNP-4_1__–__11_), at least in its linear form. To this end, *Hu* and colleagues recently observed some dependency of specific residues post position 11 in the fully folded native peptide (Hu et al., 2019). Pointing further toward enhanced bactericidal efficacy of this linear fragment, HNP-4_1__–__11__mod_, which is expected to exhibit increased stability over the non-modified version, was superior to both HNP-4_fl_ and HNP-4_1__–__11_ with a MIC several fold lower than the one observed for the natural occurring full length peptide. Additionally, we performed a time-kill assay to investigate the efficacy of HNP-4_1__–__11_ and HNP-4_1__–__11__mod_ compared to the HNP-4_fl_. Although we observed a higher potency of HNP-4_1__–__11_, the efficacy was similar to HNP-4. In contrast, HNP-4_1__–__11__mod_ was superior in both aspects (Figure 3B).

### *In vitro* Stability of HNP-4_1__–__11_ and HNP-4_1__–__11__mod_

**FIGURE 4:**
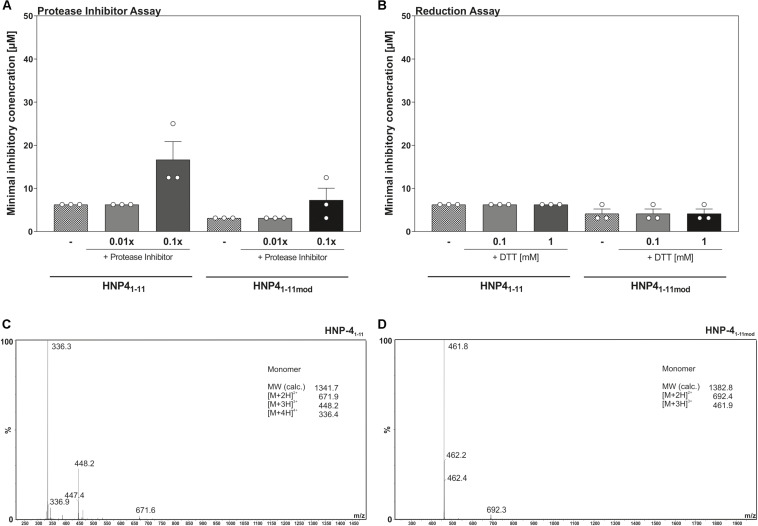
Reduction as well as proteolysis of HNP-4_1__–__11_ and HNP-4_1__–__11__mod_ have no influence on the antimicrobial activity. **(A)** Changes in the antimicrobial activity against *E. coli* ATCC25923 were analyzed in the presence of a protease inhibitor cocktail. **(B)** The minimal inhibitory concentration of HNP-4_1__–__11_ and HNP-4_1__–__11__mod_ was determined against *E. coli* ATCC25922 under reducing conditions due to the optical density after 12 h. Results from three independent experiments with ±SEM are represented. **(C)** ESI-MS analysis of HNP-4_1__–__11_ to detect potential dimer’s after peptide dilution. **(D)** Analysis of HNP-4_1__–__11__mod_ using ESI-MS to detect potential dimer’s after peptide dilution.

We modified the turbidity broth assay to determine the stability and potential resistance against proteolysis and/or natural degradation. To this end, we determined the antimicrobial activity of HNP-4_1__–__11_ and HNP-4_1__–__11__mod_ against *E. coli* ATCC25922 in presence a protease inhibitor cocktail (Figure 4A). Increasing amounts of protease inhibitors did not improve the bactericidal potential of any of the tested fragments, indicating bacterial proteases do not further degrade mentioned fragments, hence corroborating their stability. Instead, the data points toward a potential fragment:protease interaction, as high concentrations of protease inhibitors reduced the bactericidal efficacy of both fragments.

Enhanced prevalence of cysteine residues on most HDPs led to the current models of multimer formation, combined with a high net charge, as a mechanism to interact with the surface of microorganisms (Brogden, 2005; Mukherjee and Hooper, 2015). To address if multimers were essential for bactericidal efficacy, we determined the MIC of HNP-4_1__–__11_ and HNP-4_1__–__11__mod_ against *E. coli* ATCC25922 in the presence of increasing levels of the reducing agent, DTT (Figure 4B). Elevated DTT concentrations did not affect antimicrobial activity of neither HNP-4_1__–__11_ nor HNP-4_1__–__11__mod_, suggesting that monomeric peptides were sufficient to kill *E. coli* ATCC25922. To further substantiate these observations, we next performed a HPLC-MS analysis to determine possible inter-/intramolecular dimer formation (Figures 4C,D). In line with the results from our reduction assay, we did not detect any formation of oligomeric or polymeric peptide fragments.

### Cytotoxic and Hemolytic Effects of HNP-4_1__–__11_ and HNP-4_1__–__11__mod_

To determine the potential of HNP-4_1__–__11_ and HNP-4_1__–__11__mod_ for *in vivo* applications as therapeutic agents, we used two different cell lines to investigate their cytotoxic abilities.

**FIGURE 5:**
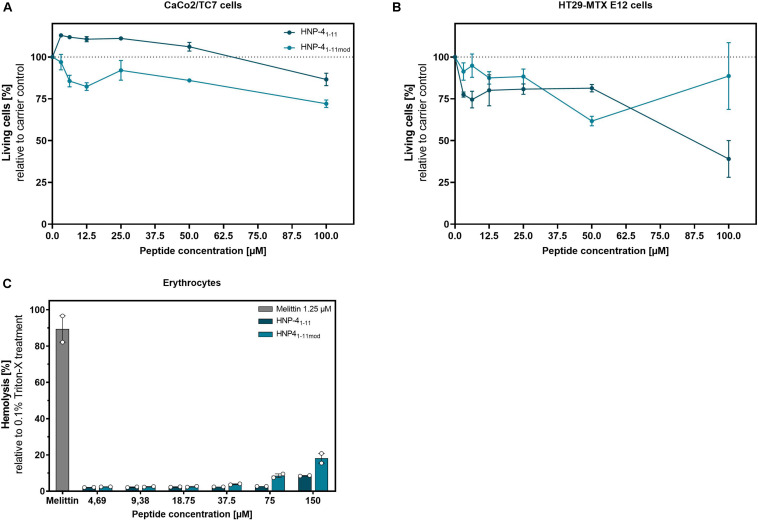
HNP-4_1__–__11_ and HNP-4_1__–__11__mod_ show only minor cytotoxic and hemolytic activity at high concentrations. We investigated the cytotoxic activity of HNP-4_1__–__11_ and HNP-4_1__–__11__mod_ against **(A)** CaCo2/TC7 or **(B)** HT29 MTX E 29 cells. We seeded 1500 cells per well and treated them after 24 h with different peptide concentrations. Living cells were determined after 96 h treatment using a CellTiter Glo2.0 assay. **(C)** Hemolytic activity on human erythrocytes of the peptides compared to 0.1% Triton-X treatment. **(A,B)** Results from three independent experiments with ±SEM are shown, and **(C)** Results from two independent experiments with ±SEM are represented.

While we only observed minor cytotoxic effects on CaCo2/TC7 cells at higher peptide concentration (Figure 5A), HT29 MTX E29 cells were more susceptible to both peptide-derivates (Figure 5B). Importantly, at lower concentrations (e.g., 12.5 μM, where HNP-4_1__–__11__mod_ has a strong antibacterial effect), the fragments exhibited only modest cytotoxicity. We additionally examined the hemolytic activity of said peptides (Figure 5C).

While HNP-4_1__–__11__mod_ has a 20% hemolytic effect at 150 μM (by far exceeding the highest concentration needed for bactericidal efficacy) there was negligible toxicity at ≤18.75 μM, i.e., the highest biological relevant concentration. Thus, compared to the honey bee toxin, Melittin, which showed an 80% hemolytic effect at 1.25 μM both HNP-4_1__–__11_ and HNP-4_1__–__11__mod_ appeared with low hemolytic activity. In conclusion, the cytotoxic concentrations identified were magnitudes higher than the corresponding bactericidal concentration.

## Discussion

Loss of antibiotic efficacy causes increased number of hospitalizations, treatment failures and spread of drug-resistant pathogens (Martens and Demain, 2017). WHO called out to develop new strategies against Gram-negative bacteria in general, and in particularly those from the WHO priority list (Tacconelli et al., 2018). To meet this request, alternatives to conventional antibiotics are urgently needed (Ghosh et al., 2019; Theuretzbacher et al., 2019, 2020). Thus, new strategies, including those of antimicrobial peptide-derivates must, be developed in the battle against multi-drug resistant bacteria (Fosgerau and Hoffmann, 2015; Breij et al., 2018). To this end proteolysis of HD-5 generated various antimicrobial active peptides with selectivity to certain bacteria (Ehmann et al., 2019). These fragments possess abilities to shift microbiota composition without decreasing diversity. Moreover, mice treated with HD-5_1__–__9_, the most potent fragment identified, harbored an increased amount of *Akkermansia* sp. (Ehmann et al., 2019). The same could be shown for the human β-defensin 1, where digestion also led to a diverse set of biological active antimicrobial fragments (Wendler et al., 2019). This study complements our earlier reports with the discovery that proteolytic digestion of HNP-4 led to a highly active easy-to-produce 11 amino acids short fragment (HNP-4_1__–__11_) with a broad antimicrobial spectrum against Gram negative and Gram positive bacteria. We hypothesize that this interesting phenomenon represents a general feature of HDPs rather than being specific to HNP-4, in part based on the observation that also the N-terminal part of HNP-1 is antimicrobial active (Varkey and Nagaraj, 2005). It is thus possible that this method of tryptic digestion of HNP-4 may be used as a general technique to unleash the antimicrobial potential of endogenous expressed HDPs to aid curbing the antibiotic resistance crises.

Interestingly, HNP-4_1__–__11_ possesses equal or better antimicrobial activity against bacteria than the full-length peptide on molar level. A modified version of this fragment further improved both potency and efficacy. Remarkably, HNP-4_1__–__11__mod_ was highly effective *in vitro* against various multidrug-resistant bacteria including *A. baumannii* 4-MRGN, *K. pneumoniae* 3-MRGN and *P. aeruginosa* 4-MRGN; all top “members” of the WHO priority and Centers for Disease Control and Prevention lists (Tacconelli et al., 2018; CDCP, 2019). Lending credence to the hypothesis of modified HDPs representing an underexplored plethora of drug candidates against multi-drug-resistant bacteria, a recent study elegantly corroborated that this exact class of bacteria are more susceptible to HDPs (Lázár et al., 2018), hence stressing their potential as new therapeutic agents. While we were able to show that HNP-4_1__–__11_ and HNP-4_1__–__11__mod_ displayed a broad spectrum antimicrobial activity pattern, we did not focus on their antimicrobial mechanisms, but the capacity to induce rapid killing of Gram-negative bacteria indicates membrane interactions as part of the mode(s) of action. From a general point of view cysteines and charged amino acids are often relevant for antimicrobial activity (Jiang et al., 2008). Importance of those amino acids led to the current models of HDP mechanism forming multimers as well as the need of charged amino acids to interact with the surface of microorganisms (Brogden, 2005; Mukherjee and Hooper, 2015). Due to these observations, we initially assumed that also the antimicrobial activity of the here presented fragments depended on dimerization. Yet, our reducing assays followed by HPLC-MC analysis illustrated that monomeric formation was sufficient for the observed bactericidal activity, pointing toward a different mode of action of these hallmark peptide fragments, disputing the current dogma in the field.

Although covalent dimers are absent, non-covalent oligomeric forms of both peptides cannot be entirely excluded. Additional analyses are necessary to determine the importance of supramolecular peptide forms for antimicrobial activity, as non-covalent oligomerization can be relevant for antimicrobial activity of several and in particular amyloid-forming peptides (Latendorf et al., 2019).

A challenge with HDPs in therapeutic contexts is their susceptibility to proteolysis by bacterial proteolytic enzymes (Reijmar et al., 2007), in particular in reduced environments (Schroeder et al., 2011a), as exemplified by the outer membrane protease of *Salmonella enterica* which degrades and thereby inactivates HDPs, thus supporting an essential role of bacterial proteases in bacterial resistance to HDPs (Guina et al., 2000). The conceptual advancement of utilizing protease-degraded biologically active fragments, as showcased here by trypsin digest is therefore intriguing. Such fragments should, by nature, be resistant to further degradation and may prove valuable to aid fight multi-drug resistant pathogens. In keeping with this notion, our analysis revealed that HNP-4_1__–__11_ and HNP-4_1__–__11__mod_ activity was not further boosted by protease inhibitors, suggesting that proteases *per se* do not hamper their function. Instead, high levels of protease inhibitors appeared to limit the bactericidal efficacy of both HNP-4_1__–__11_ and HNP-4_1__–__11__mod_ suggesting that these fragments conversely interact with proteases, rather than being annulled by them, to induce bacterial killing. Future studies are warranted to elucidate the extent of such potential fragment:protease interaction.

For potential therapeutic application, we assessed toxicity of HNP-4_1__–__11_ and HNP-4_1__–__11__mod_. Both peptides showed cell-type dependent cytotoxicity and hemolytic activity at higher concentrations. To this end, HNP-4_1__–__11__mod_ exerted a greater impact on CaCo-2 cells, whereas HNP-4_1__–__11_ possessed higher cytotoxicity against HT29-MTX E12 cells, but for both tested cell types the cytotoxic concentration range were magnitudes higher than the concentrations needed for antimicrobial activity.

In summary, although future *in vivo* experiments are warranted to determine the full potential of HNP-4_1__–__11_ and HNP-4_1__–__11__mod_, our results demonstrate promising efficacy of HNP-4_1__–__11_ and HNP-4_1__–__11__mod_ against multidrug-resistant bacteria. From this point of view, proteolytic digestion of HDPs could be used to generate new biologically active fragments to overcome the antibiotic-resistance crisis.

## Data Availability Statement

The datasets generated for this study are available on request to the corresponding author.

## Ethics Statement

The study protocol was previously approved by the Ethical Committee of the University Hospital Tübingen, Germany. Patients and controls who were included in this study all gave their written and informed consent after the study purpose, samples procedure, and potential adjunctive risks were explained. All experiments were conducted in accordance with the relevant guidelines and regulations.

## Author Contributions

DE, LK, and JaW designed the study. DE, LK, and JuW performed the experiments. DE, LK, BJ, and JaW analyzed the data. DE, LK, JaW, and BJ wrote the manuscript. JuW and NM assisted with data interpretation and manuscript editing. JaW and BJ supervised all parts of the study. All authors were involved in data discussion and approved the final version of the manuscript.

## Conflict of Interest

The authors declare that the research was conducted in the absence of any commercial or financial relationships that could be construed as a potential conflict of interest.

## Supplementary Material

FIGURE S1 RDA with the HNP-4 fragments against commensal bacteria. Here we show the detailed results of the RDA experiments. Data are presented as mean ± SEM. Experiments were carried out three independent times.

FIGURE S2 RDA with the HNP-4 fragments against pathogenic bacteria. Here we show the detailed results of the RDA experiments. Data are presented as mean ± SEM. Experiments were carried out three independent times.

FIGURE S3 Analytical data sheet of HNP-4_1__–__11_ and HNP-4_1__–__11__mod_. Here we show the detailed analysis of purity of HNP-4_1__–__11_ and HNP-4_1__–__11mod_.
