# Supplementary figures and images for: Fragmentation of Human Neutrophil α-Defensin 4 to Combat Multidrug Resistant Bacteria
Source: Front Microbiol. 2020 Jun 3;11:1147. doi: 10.3389/fmicb.2020.01147 (PMC7286198; doi:10.3389/fmicb.2020.01147)

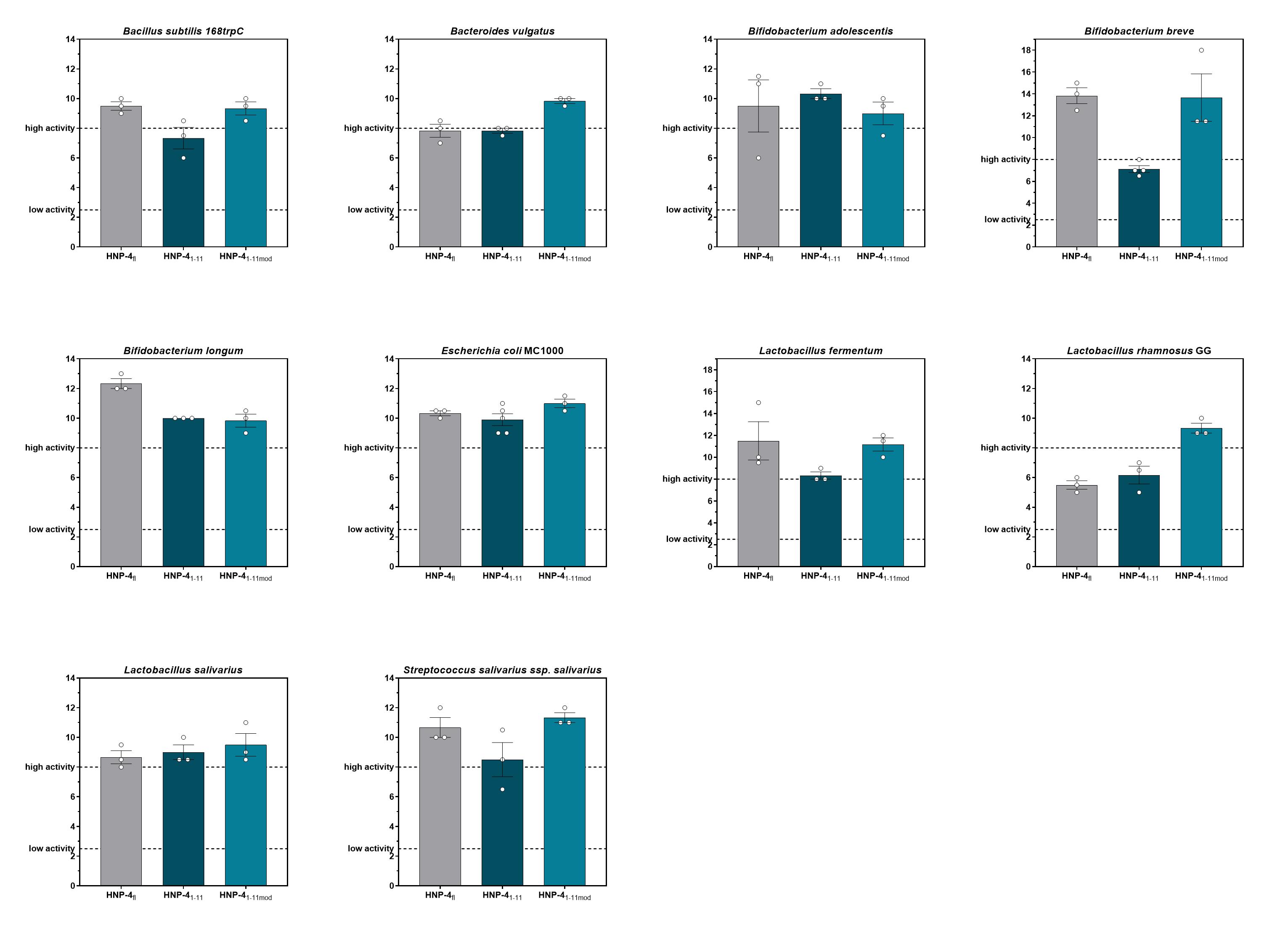

Supplement: FIGURE S1 — RDA with the HNP-4 fragments against commensal bacteria. Here we show the detailed results of the RDA experiments. Data are presented as mean ± SEM. Experiments were carried out three independent times. [file Image_1.JPEG]

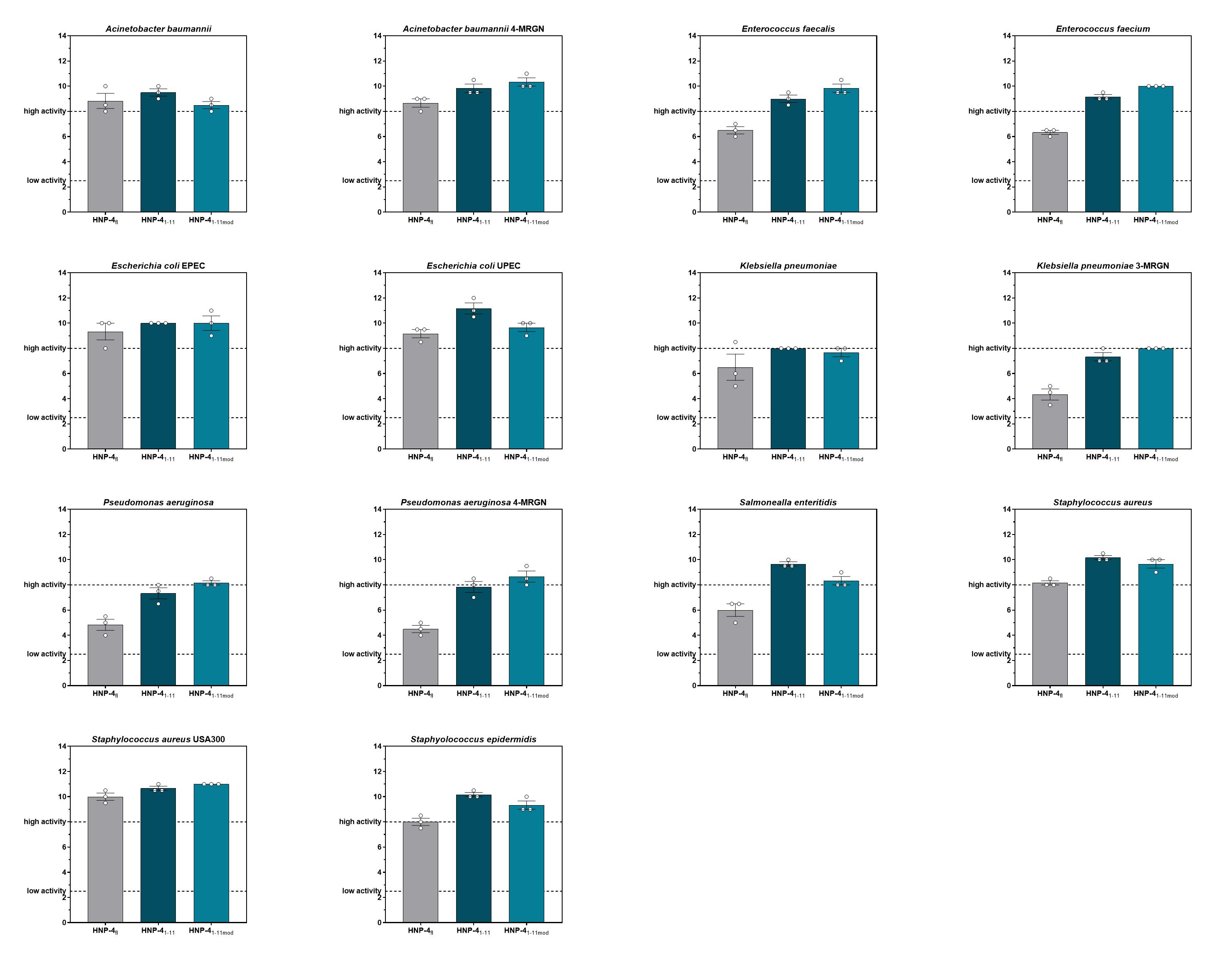

Supplement: FIGURE S2 — RDA with the HNP-4 fragments against pathogenic bacteria. Here we show the detailed results of the RDA experiments. Data are presented as mean ± SEM. Experiments were carried out three independent times. [file Image_2.JPEG]

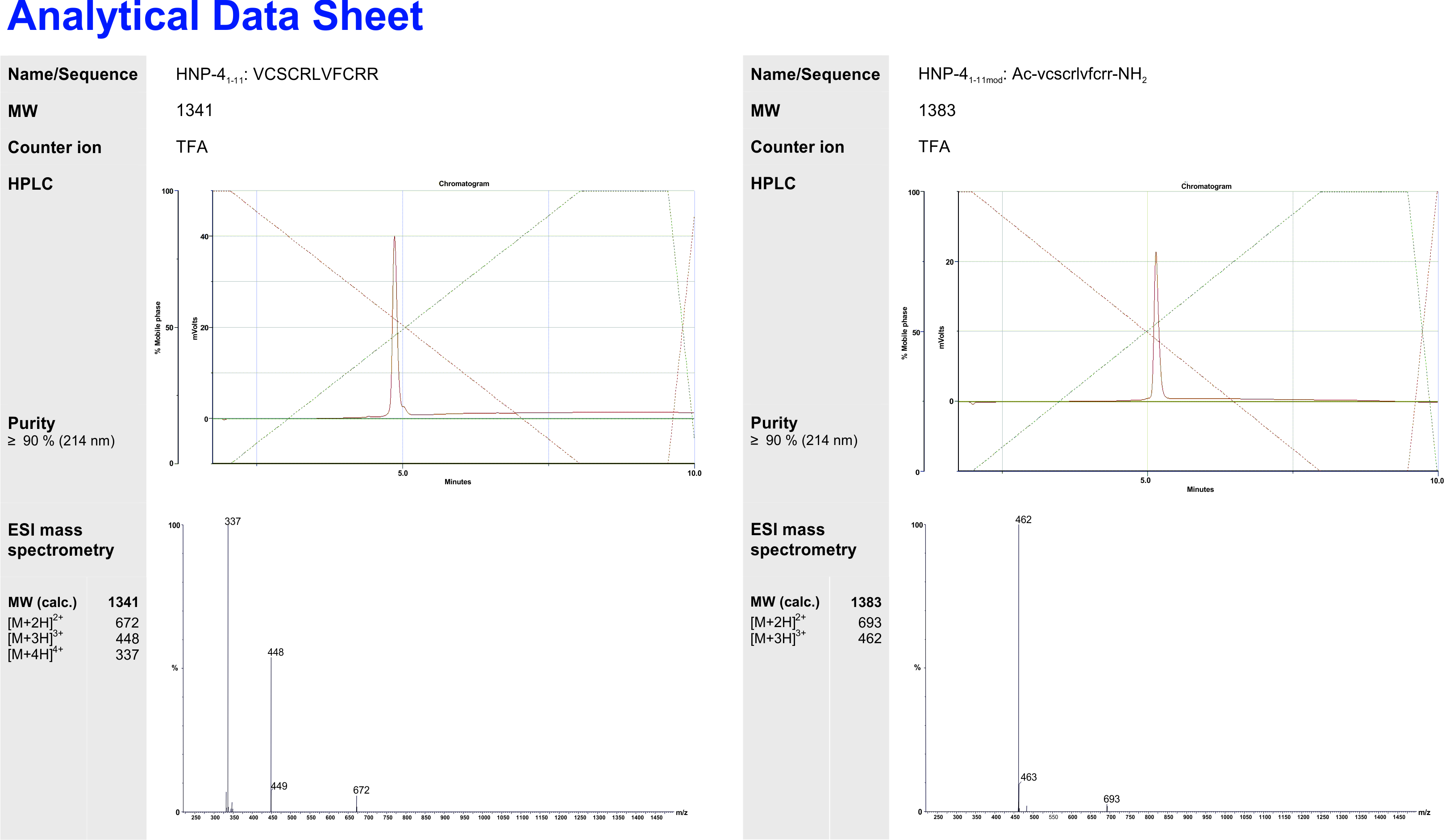

Supplement: FIGURE S3 — Analytical data sheet of HNP-41–11 and HNP-41–11mod. Here we show the detailed analysis of purity of HNP-41–11 and HNP-41–11mod. [file Image_3.JPEG]
